# The Unique Glycosylation at Position 986 on the E2 Glycoprotein of Classical Swine Fever Virus Is Responsible for Viral Attenuation and Protection against Lethal Challenge

**DOI:** 10.1128/JVI.01768-21

**Published:** 2022-01-26

**Authors:** Yongfeng Li, Mengqi Yuan, Yuying Han, Libao Xie, Yuteng Ma, Su Li, Yuan Sun, Yuzi Luo, Weike Li, Hua-Ji Qiu

**Affiliations:** a State Key Laboratory of Veterinary Biotechnology, Harbin Veterinary Research Institute, Chinese Academy of Agricultural Sciences, Harbin, China; b State Key Laboratory of Veterinary Etiological Biology, Lanzhou Veterinary Research Institute, Chinese Academy of Agricultural Sciences, Lanzhou, China; Instituto de Biotecnologia/UNAM

**Keywords:** classical swine fever virus, C-strain, glycosylation, attenuation, protection

## Abstract

Classical swine fever (CSF) is an economically important disease of pigs caused by classical swine fever virus (CSFV). The live attenuated vaccine C-strain (also called HCLV strain) against CSF was produced by multiple passages of a highly virulent strain in rabbits. However, the molecular determinants for its attenuation and protection remain unclear. In this study, we identified a unique glycosylation at position 986 (^986^NYT^988^) on the E2 glycoprotein Domain IV of C-strain but not (^986^NYA^988^) the highly virulent CSFV Shimen strain. We evaluated the infectivity, virulence, and protective efficacy of the C-strain-based mutant rHCLV-T988A lacking the glycosylation and Shimen strain mutant rShimen-A988T acquiring an additional glycosylation at position 986. rShimen-A988T showed a significantly decreased viral replication ability in SK6 cells, while rHCLV-T988A exhibited a growth kinetics indistinguishable from that of C-strain. Removal of the C-strain glycosylation site does not affect viral replication in rabbits and the attenuated phenotype in pigs. However, rShimen-A988T was attenuated and protected the pigs from a lethal challenge at 14 days postinoculation. In contrast, the rHCLV-T988A-inoculated pigs showed transient fever, a few clinical signs, and pathological changes in the spleens upon challenge with the Shimen strain. Mechanistic investigations revealed that the unique glycosylation at position 986 influences viral spreading, alters the formation of E2 homodimers, and leads to increased production of neutralizing antibodies. Collectively, our data for the first time demonstrate that the unique glycosylation at position 986 on the E2 glycoprotein is responsible for viral attenuation and protection.

**IMPORTANCE** Viral glycoproteins involve in infectivity, virulence, and host immune responses. Deglycosylation on the E^rns^, E1, or E2 glycoprotein of highly virulent classical swine fever virus (CSFV) attenuated viral virulence in pigs, indicating that the glycosylation contributes to the pathogenicity of the highly virulent strain. However, the effects of the glycosylation on the C-strain E2 glycoprotein on viral infectivity in cells, viral attenuation, and protection in pigs have not been elucidated. This study demonstrates the unique glycosylation at position 986 on the C-strain E2 glycoprotein. C-strain mutant removing the glycosylation at the site provides only partial protection against CSFV challenge. Remarkably, the addition of the glycan to E2 of the highly virulent Shimen strain attenuates the viral virulence and confers complete protection against the lethal challenge in pigs. Our findings provide a new insight into the contribution of the glycosylation to the virus attenuation and protection.

## INTRODUCTION

Classical swine fever (CSF) is an economically important disease of pigs in many countries. The etiologic agent classical swine fever virus (CSFV) belongs to the *Pestivirus* genus within the *Flaviviridae* family (1, 2). CSFV contains a single-stranded, positive-sense RNA genome consisting of a 5′-untranslated region (5′-UTR), a single large open reading frame (ORF), and a 3′-UTR. The ORF encodes a polyprotein that, upon proteolytic processing, gives rise to 12 proteins in the following order: NH_2_-N^pro^-C-E^rns^-E1-E2-p7-NS2-NS3-NS4A-NS4B-NS5A-NS5B-COOH (3, 4).

The CSFV C-strain, also known as hog cholera lapinized virus (HCLV) strain, is considered one of the best modified live vaccines against CSF. It has contributed greatly to the global control and eradication of CSF (5). C-strain is characterized by a typical fever response in the rabbit that lasts 18–24 h. Furthermore, robust viral replication in the spleen represents the adaptation of the C-strain to the rabbit. Our recent study demonstrated that C-strain with the 3′-UTR replacement by that of the highly virulent CSFV Shimen strain lost its ability of fever induction but retained its replication in the spleen and immunogenicity in rabbits (6). This vaccine provides complete protection for pigs of almost all ages against lethal challenge (7). However, the molecular determinants of C-strain attenuation and protection in pigs remain unexplored.

Glycosylation on the viral envelope glycoproteins plays a critical role in viral pathogenesis (8, 9). It has been demonstrated that glycosylation status on the E^rns^, E1, or E2 glycoproteins of the highly virulent CSFV strain Brescia affects virulence in pigs (10–12). Removal of *N*-linked glycosylation on the hemagglutinin-neuraminidase protein of Newcastle disease virus renders an attenuated virus in chickens (13). Similarly, the hemagglutinin (HA) glycosylation patterns of highly pathogenic avian influenza virus H5N1 are associated with the virulence in chickens (14). A recent study has shown that the envelope protein glycosylation is critical for Zika virus (ZIKV) infection of mammalian and mosquito hosts (15).

Glycosylation contributes to the immunogenicity of viral envelope glycoproteins (16–20). Glycan repositioning of the influenza hemagglutinin stem facilitates cross-subtype protective antibody responses (20). It has been shown that glycosylation of the GP5 glycoprotein of porcine respiratory and reproductive syndrome virus affects viral antigenicity and ability to induce neutralizing antibodies (NAbs) in pigs (16). Altered glycosylation patterns increase the immunogenicity of a hepatitis C subunit vaccine, inducing NAbs in mice (18). The CSFV E2 glycoprotein is exposed to the viral outer surface and is the most immunodominant protein inducing the major NAbs in infected pigs (21). Glycans have also been shown to play a key role in immune evasion through masking antigenic sites targeted by NAbs (22–24). The addition of two glycosylation sites at positions 135 and 160 in the HA protein facilitated the escape of the H7N9 avian influenza viruses from the vaccine-induced immunity (24).

This study aimed to clarify the effects of the unique glycosylation at position 986 on the E2 glycoprotein Domain IV of C-strain on viral infectivity in cells, viral attenuation, protection, and antigenicity in pigs.

## RESULTS

### The E2 glycoprotein of the C-strain contains a unique glycosylation at position 986.

**FIG 1:**
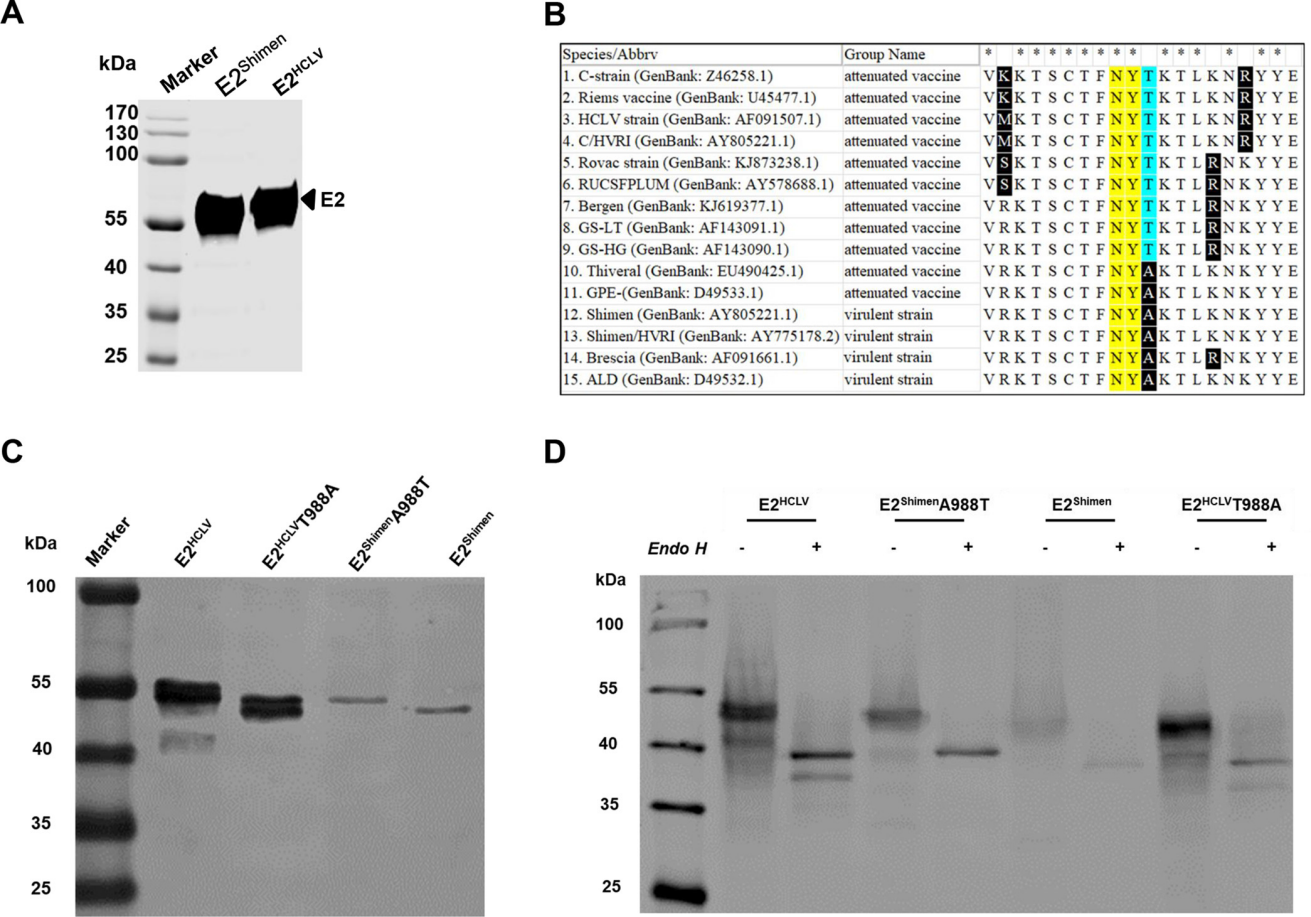
Identification of the unique glycosylation at position 986 (^986^NYT^988^) on the E2 glycoprotein of the C-strain. (A) Analysis of the E2 glycoprotein of highly virulent CSFV Shimen strain (E2^Shimen^) or C-strain (E2^HCLV^) by Western blotting using the anti-E2 MAb WH303. (B) A multiple alignment of the 20 amino acids surrounding the *N*-glycosylation at position 986 is present for several CSFV strains of known virulence. (C) Mobility of E2^Shimen^A988T harboring the mutation A988T and E2^HCLV^T988A containing the mutation T988A analyzed by Western blotting. (D) Analysis of the E2^HCLV^ and E2^Shimen^ treated or not with endoglycosidase H by Western blotting.

Although the E2 glycoprotein of either the highly virulent Shimen strain (E2^Shimen^) or live attenuated vaccine C-strain (E2^HCLV^) has same numbers of the amino acid residues, their mobility is different (Fig. 1A). We assumed that the glycosylation could contribute to the difference. Five potential *N*-linked glycosylation residues were observed on the E2^Shimen^, while six sites were found on E2^HCLV^, which has the unique glycosylation at position 986 in the sequence motif ^986^NYT^988^. Furthermore, sequence alignment showed that the E2 glycoproteins from vast majority of attenuated CSFV strains (9/11) contain the *N*-linked glycosylation motif (N-X-T/S) (Fig. 1B).

In addition, E2^Shimen^A988T with the A988T mutation acquired the glycosylation at position 986, while E2^HCLV^T988A containing the T988A mutation lost the glycosylation at the site (Fig. 1C). After treatment with the endoglycosidase H, the mobility of E2^HCLV^ glycoprotein evolved into the same as that of the E2^Shimen^, indicating that the mobility difference is due to the different degrees of glycosylation (Fig. 1D).

### Generation of the mutants with altered glycosylation in the background of the C-strain or the Shimen strain.

**FIG 2:**
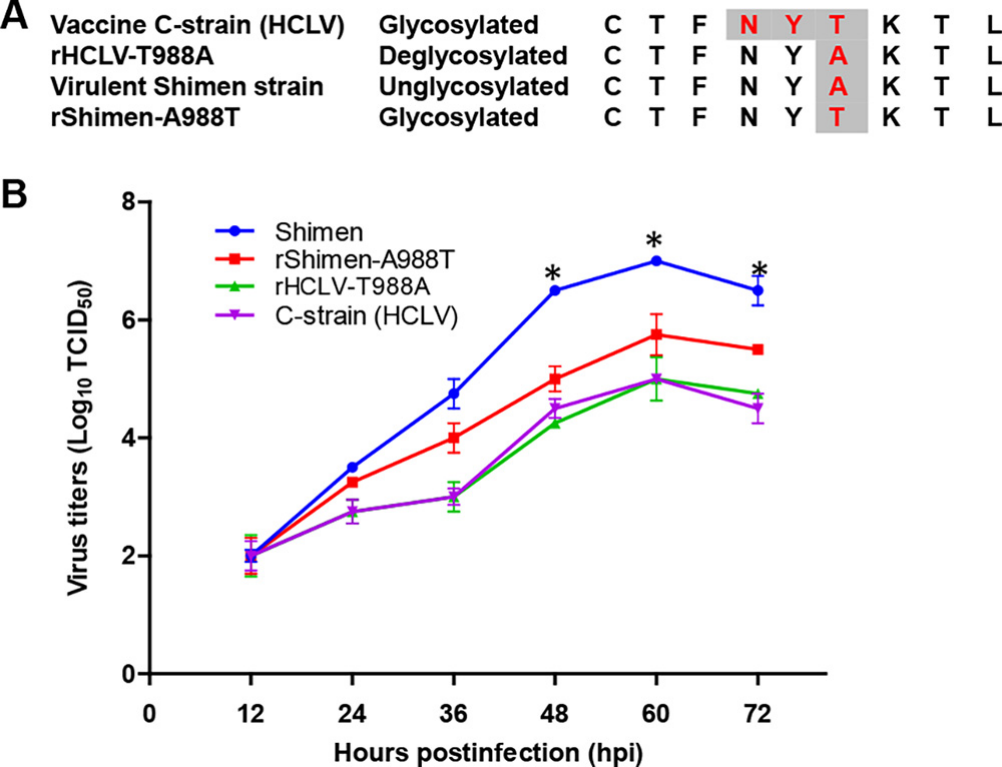
Generation and characterization of the CSFV mutants rHCLV-T988A and rShimen-A988T. (A) Amino acid sequence of the parental viruses and the mutant viruses around the ^986^NYT^988^ motif and E2 glycosylation status of parental viruses and mutants. C-strain-based rHCLV-T988A lacks the glycosylation by mutation T988A in the motif ^986^NYT^988^ and the Shimen strain mutant rShimen-A988T acquires the glycosylation at position 986 by mutation A988T in its motif ^986^NYA^988^. (B) *In vitro* growth characteristics of glycosylation mutants and parental C-strain and the highly virulent Shimen strain in SK6 cells. The error bars represent the standard deviations for three replicates. *, *P < *0.05 (Student's *t* test).

To examine the roles of this *N*-linked glycosylation, we generated two mutant viruses by exchanging the T and A at position 988 in the motif. Briefly, glycosylation at position 986 is eliminated by the mutation T988A to generate a C-strain-based mutant rHCLV-T988A. In the backbone of the Shimen strain, the glycan is added by mutating A988T, creating rShimen-A988T. The glycosylation status and sequences of the E2 glycoprotein of mutant viruses compared to that of the parental viruses around this site were shown in Fig. 2A The two mutants were recovered by transfection of individual infectious clones into SK6 cells, and the transfected cells were serially passaged. Indirect-immunofluorescence assay (IFA) revealed that the two mutants were successfully rescued and were infectious (data not shown). Full-length clones and *in vitro*-rescued viruses were completely sequenced. Sequencing of the full genome of rescued viruses confirmed the engineered mutation without reversion or other mutations.

The growth characteristics of the mutants were determined in SK6 cells. The results showed that the titers of rShimen-A988T were significantly decreased compared with the Shimen strain (*P < *0.05), while the rHCLV-T988A exhibited indistinguishable growth kinetics from that of parental C-strain (Fig. 2B).

### The T988A mutation in C-strain does not affect viral replication in rabbits.

Rabbit inoculation experiments were conducted to analyze whether the ^986^NYT^988^ on the E2 glycoprotein of C-strain affects its characteristics in the rabbit. Three groups of 14 rabbits were inoculated with the mutant rHCLV-T988A or parental virus, according to Table 1. Rectal temperature was recorded before inoculation and every 6 h from 24 to 72 hours postinoculation (hpi). According to the fever response standard described previously (6), rHCLV-T988A did not induce fever response in rabbits in contrast to C-strain (Table 1). Two or three rabbits in each group were randomly euthanized to determine the viral genome copies in the spleens by quantitative reverse transcription-PCR (RT-qPCR) (25). The results showed that viral genome was detected in the groups inoculated with rHCLV-T988A or C-strain but not DMEM, indicating that abrogating the glycosylation of C-strain does not affect its replication in the rabbits but disable the fever response capacity of C-strain in rabbits.

**TABLE 1:** Viral replication in the spleens of the rabbits inoculated with the C-strain-based mutant and HCLV

Inocula	Dose (TCID_50_)	Fever induced	No. of viral replication/total	Mean viral RNA copies in the spleens (copies/μl)	Seroconversion at 10 dpi
rHCLV-T988A	10^4^	0/6	3/3	5.00 × 10^3^	3/3
HCLV	10^4^	4/4	2/2	5.24 × 10^3^	2/2
DMEM	1 ml	0/4	0/2	No Ct	0/2

### The glycosylation at position 986 affects virus spreading in cells.

**FIG 3:**
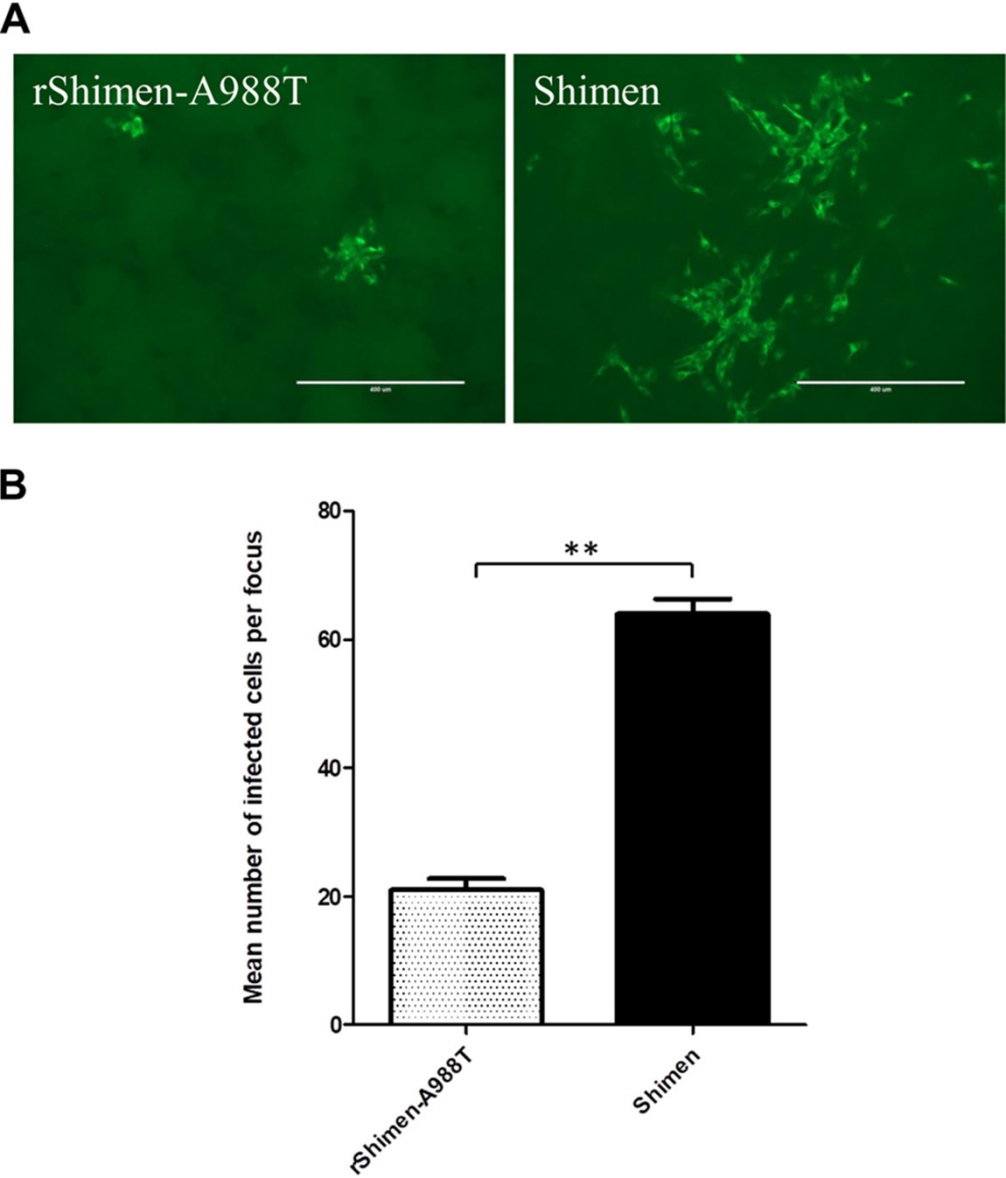
Virus spreading in cell culture infected by the mutant rShimen-A988T and the parental virus. (A) PK-15, SK6 cells, or swine macrophages were inoculated at an MOI of 0.001 with the parent and mutant viruses as indicated. The cell monolayers were overlaid with DMEM containing 1% Bacto agar and incubated at 37°C in the presence of 5% CO_2_. After 48-h incubation, the cells were fixed with cold acetone, and viral E2 was examined by immunofluorescence using the anti-E2 MAb WH303. Representative focus sizes of PK-15 cells infected by the viruses are shown. (B) The infected cells per focus were counted for 30 independent foci (*n *= 30). Error bars represent the standard deviations. **, *P < *0.01 (Student's *t* test).

The replication of rShimen-A988T was significantly decreased compared to the Shimen strain as described above. Next, we detected whether the A988T mutation in E2 affected virus spreading. To determine the effects of the amino acid substitution on viral spreading, monolayers of SK6 cells, PK-15 cells, or swine macrophages were infected with the mutant rShimen-A988T or the parental virus Shimen strain, and focus formation was monitored under a semisolid overlay using viral antigen detection by immunofluorescence. The infected cells per focus were counted for 30 randomly selected foci, and the data were statistically analyzed. The mutant virus with A988T mutation produced the smallest foci (Fig. 3A), comparable in size to the foci obtained with the parental virus in the cells (Fig. 3B). These results indicate that the glycosylation on E2 is a major determinant of the virus spread in cells.

### Decreased E2 dimerization in rShimen-A988T-infected SK6 cells.

**FIG 4:**
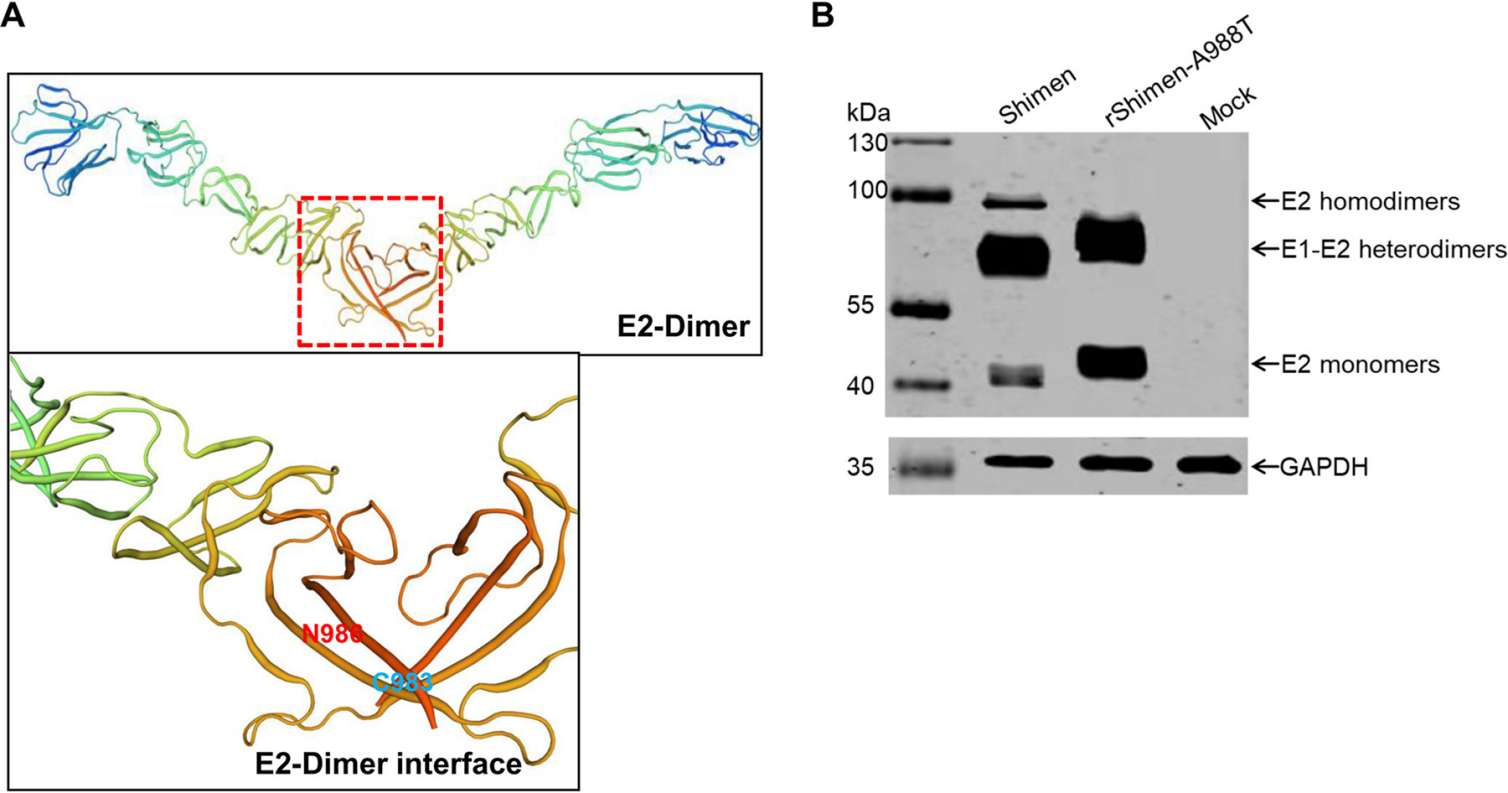
Western blotting of SK6 cell lysates infected with the mutant virus rShimen-A988T and the parental virus Shimen strain. (A) Three-dimensional structure of E2 homodimers of CSFV (Model of structure of BVDV glycoprotein E2, pH 8). The boxed region in the top diagram is enlarged below, showing the dimer interface. The glycosylation at position 986 (N986) in the dimer interface and Cys at 983 (C983) are indicated. (B) Detection of E2 homodimers and E1-E2 heterodimers with the anti-E2 MAb WH303. SDS-PAGE was performed under the nonreducing conditions. GAPDH was used as an internal control. This experiment was done three times.

The motif ^986^NYT^988^ is in close proximity to the E2 dimer interface (Fig. 4A). The conserved Cys residue at position 983 on the E2 glycoprotein is the site of disulfide bond linkage with E1 to form E1-E2 heterodimers. Actually, this Cys is in very close proximity to the ^983^CTFNYT^988^ motif harboring the *N*-linked glycosylation site. It is conceivable that the presence/absence of the *N*-glycosylation at position 986 has a significant influence on the dimerization of E2, which could explain the observed relevance of this sugar on the replication of the virus. Therefore, we investigated the effects of the mutations in the ^986^NYT^988^ motif on E2-E2 homodimerization and E1-E2 heterodimerization.

Under nonreducing conditions, the lysates from cells infected with rShimen-A988T showed changes in the proportions of expressed homo-/heterodimers as determined by Western blotting using anti-E2 monoclonal antibody (MAb) WH303 (Fig. 4B). Infection with rShimen-A988T led to a decreased formation of E2 homodimers and increased accumulation of E2 monomers relative to parental virus infection. Additionally, as expected, due to the glycosylation, the electrophoretic mobility of E1-E2 heterodimers and E2 monomers of rShimen-A988T was slower than that of the parental Shimen strain. The data confirm that the *N*-glycosylation at position 986 affects the formation of E2 homodimers.

### The Shimen strain-based mutant with the A988T mutation is attenuated in pigs.

**FIG 5:**
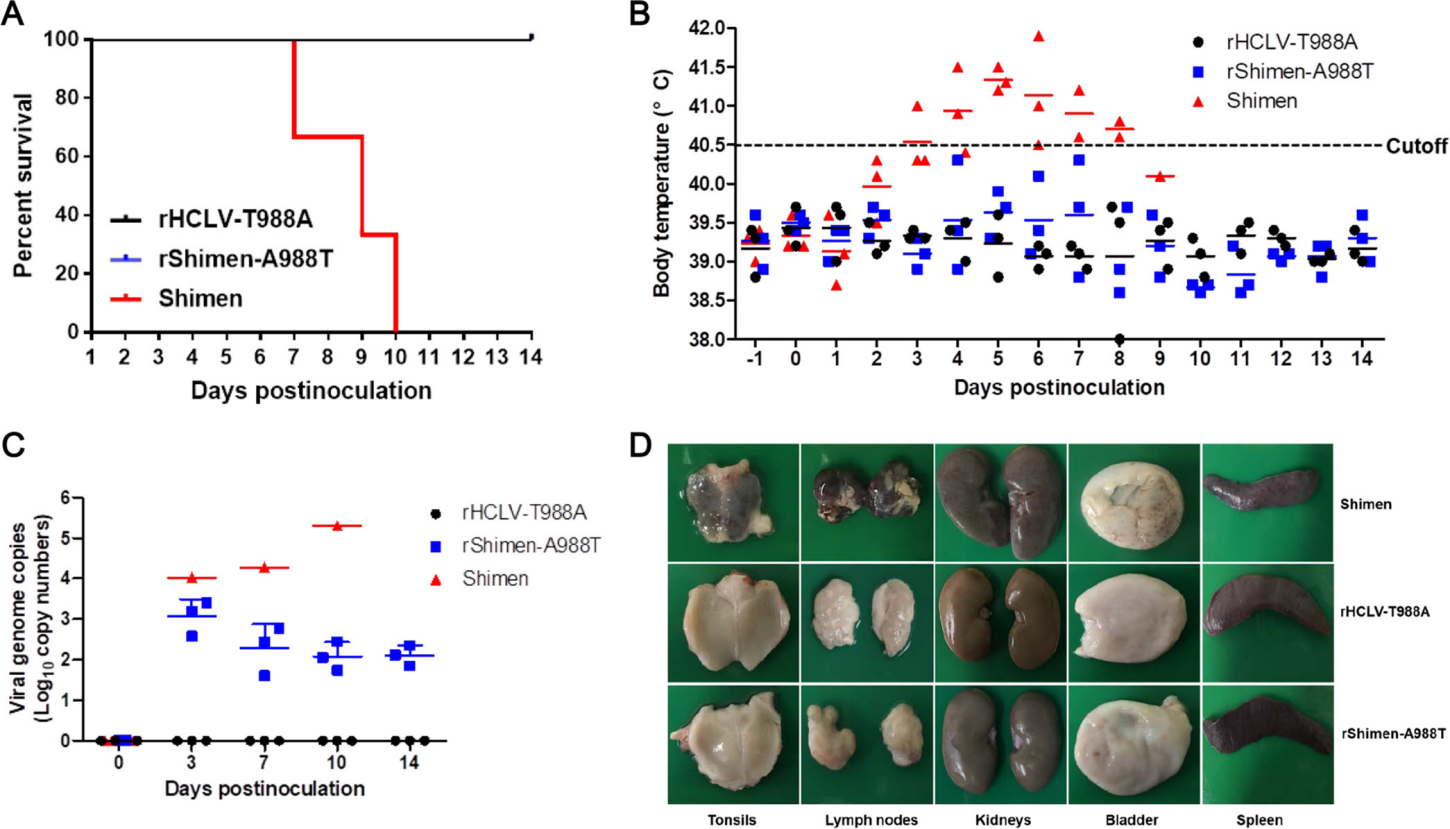
The Shimen strain mutant with the A988T mutation is attenuated in pigs. (A) Mortality of the pigs inoculated with 10^4^ TCID_50_ of parental virus Shimen strain, rHCLV-T988A, or rShimen-A988T, respectively. The pigs in group 1 were inoculated intramuscularly (i.m.) with 10^4^ TCID_50_ of rHCLV-T988A, the pigs in group 2 were inoculated i.m. with 10^4^ TCID_50_ of rShimen-A988T, and the pigs in group 3 were infected with the Shimen strain as positive control to assess the pathogenicity. (B) Rectal temperatures of the pigs inoculated with different viruses are shown at the indicated days postinoculation. (C) The viral genome copy numbers in the blood of the pigs inoculated with mutants or the parental virus were tested by RT-qPCR at the indicated days postinoculation. (D) Representative pathological changes of various organs from the pigs inoculated with the parental virus Shimen strain, rHCLV-T988A, or rShimen-A988T.

Since glycosylation of the viral envelope glycoproteins affects viral pathogenesis, we conducted *in vivo* experiments to clarify whether the introduction of glycosylation at position 986 to the E2 glycoprotein of the Shimen strain could affect viral virulence in pigs. Three groups of pigs were inoculated with 10^4^ TCID_50_ of parental virus Shimen strain, rHCLV-T988A, or rShimen-A988T. One pig from Shimen-infected group died at 7 days postinoculation (dpi), other pigs died at 9 dpi and at 10 dpi, respectively (Fig. 5A). The pigs inoculated with rHCLV-T988A or rShimen-A988T did not present fever responses or any clinical signs associated with CSF during 0 to 14 dpi (Fig. 5B). As expected, animals infected with the Shimen strain exhibited clinical signs associated with acute CSF, including fever (Fig. 5B).

The animals infected with rShimen-A988T present low levels of viremia, while rHCLV-T988A induced almost undetectable levels of viremia during the 14-day observation period (Fig. 5C). However, high viremia was detectable and gradually increased in anticoagulant venous blood from animals infected with the Shimen strain by RT-qPCR at 3 dpi until the animals died before 11 dpi (Fig. 5C). Furthermore, CSF-specific pathological changes were observed only in the pigs inoculated with Shimen strain but not in rHCLV-T988A or rShimen-A988T (Fig. 5D).

Therefore, the introduction of the glycan at position 986 to the E2 glycoprotein of the Shimen strain attenuates viral virulence in pigs, while losing the glycosylation at the site does not affect the attenuation phenotype of the C-strain.

### The Shimen strain-based mutant with the A988T mutation provides complete protection against lethal challenge.

**FIG 6:**
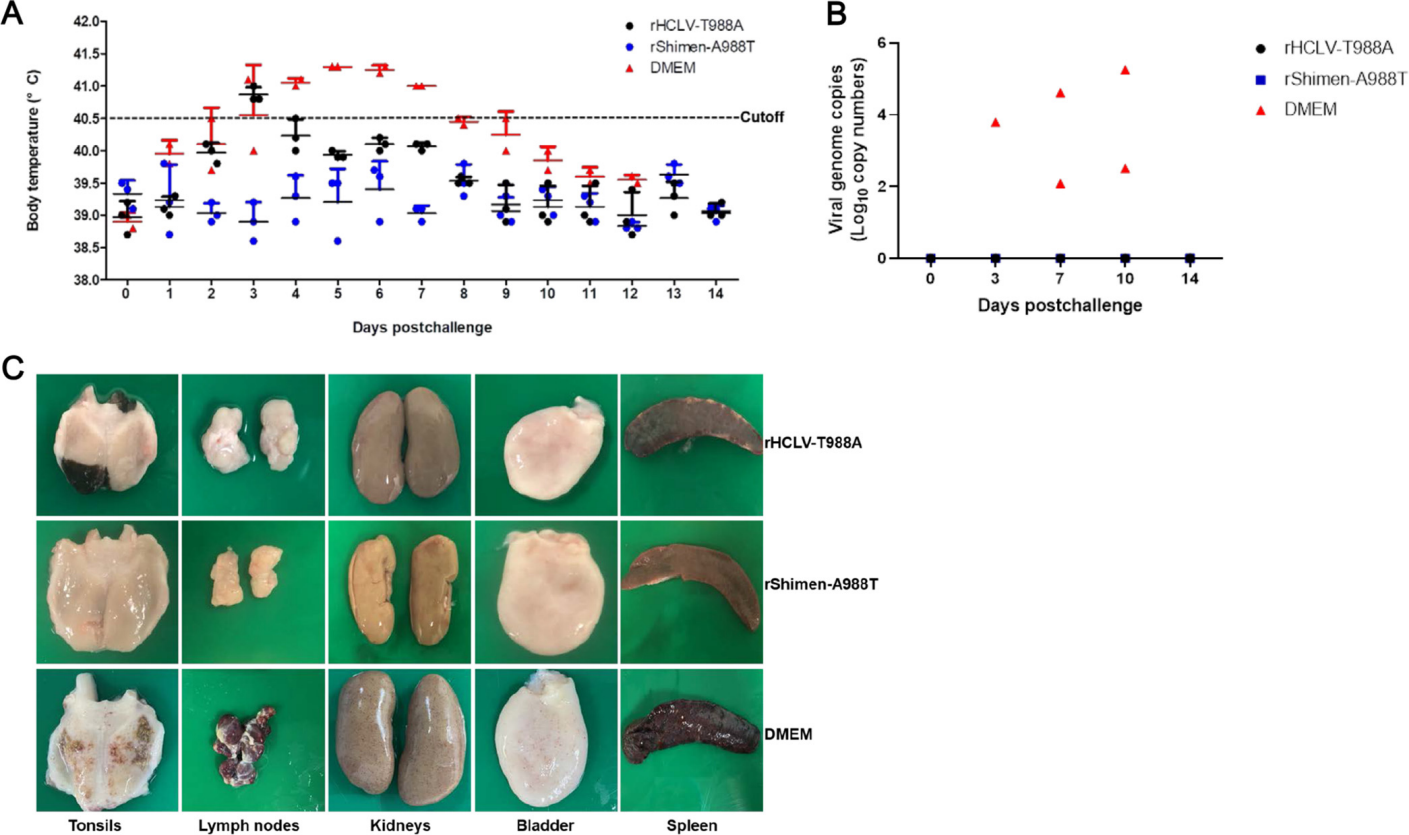
The Shimen strain with the A988T mutation provides complete protection of pigs from lethal CSFV challenge. At 14 days postinoculation, the pigs in Group A (rHCLV-T988A), Group B (rShimen-A988T), and Group C (DMEM) were challenged with 10^5^ TCID_50_ of the Shimen strain. (A) Rectal temperatures of the pigs challenged with the highly virulent Shimen strain were shown at the indicated days postchallenge (dpc). (B) The viral genome copy numbers in the blood samples of the pigs inoculated with mutants or parental virus were determined by RT-qPCR at the indicated dpc. (C) Representative pathological changes in various organs from the inoculated pigs following challenge with the Shimen strain.

The potential of rShimen-A988T to induce protection against the Shimen strain challenge was assessed. Three groups of pigs were challenged intramuscularly (i.m.) at 14 dpi with 10^5^ TCID_50_ of the Shimen strain. The three pigs inoculated with rShimen-A988T were completely protected when challenged at 14 dpi, while the pigs inoculated with rHCLV-T988A showed moderate clinical signs and pathological changes in the spleens associated with CSF following challenge (Fig. 6A). As expected, the two mock-vaccinated control pigs receiving the Shimen strain developed anorexia, depression, and fever at 3 to 4 days postchallenge (dpc) and died or were euthanized *in extremis* by 14 dpc.

Viremia in vaccinated and challenged animals was examined at different dpc. The animals inoculated with rShimen-A988T did not produce detectable viremia following the challenge with the Shimen strain (Fig. 6B). As expected, in mock-vaccinated control animals, viremia was observed at 3 dpc, with virus titers remaining typically high at the last time point tested before the animals died or euthanized. Moreover, the pigs inoculated with rShimen-A988T displayed no pathological lesions, while pathological changes were found in the spleens of the pigs inoculated with rHCLV-T988A (Fig. 6C). Throughout the time of challenge experiment, CSF-specific pathological changes were observed (including enlargement and hemorrhages in the lymph nodes, petechiae in the kidneys, and infarcts in the spleen) in the pigs inoculated with DMEM (26, 27).

Taken together, these results indicate that complete protection from CSF-related clinical signs and the replication of the challenge virus was induced by rShimen-A988T.

### Pigs immunized with rShimen-A988T develop robust NAbs.

**FIG 7:**
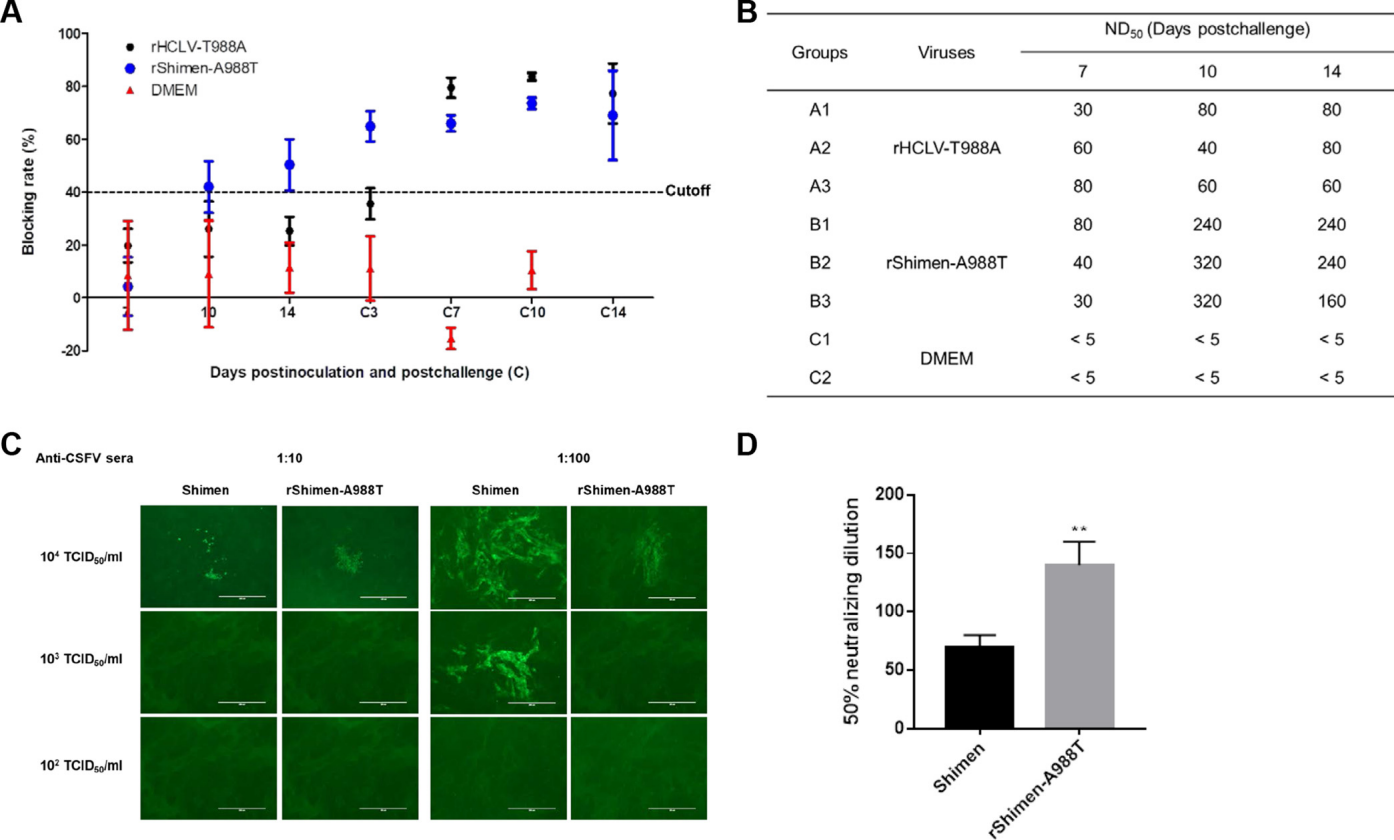
The CSFV Shimen strain with the A988T mutation is more efficient to be neutralized than the parental virus by the sera. (A) Anti-E2 antibodies in the sera from the pigs inoculated with different viruses were determined at the indicated days postinoculation and postchallenge. (B) Neutralizing antibody titers against the Shimen strain. The sera were collected from the pigs inoculated with different viruses after the challenge of highly virulent CSFV Shimen strain. (C) Comparison of neutralizing ability against rShimen-A988T and parental virus using the sera collected from the pigs inoculated with the C-strain followed by challenging with the Shimen strain. Bar, 400 μm. (D) Neutralizing titers of the same sera (*n *= 3) to neutralize each of the viruses. **, *P < *0.01 (Student's *t* test).

Since glycosylation influences the antigenicity of viral envelope glycoproteins, anti-E2 antibodies and NAbs against the CSFV Shimen strain in the sera were determined by blocking ELISA and neutralization assay, respectively. One out of three pigs inoculated with rShimen-A988T seroconverted at 10 dpi, and the anti-E2 antibodies were detected in the sera from all the inoculated pigs at 14 dpi (Fig. 7A). The results showed that higher titers of NAbs against CSFV were detected in the sera of the pigs inoculated with rShimen-A988T than that of rHCLV-T988A at 7, 10, and 14 dpc (Fig. 7B). Notably, rShimen-A988T was more efficiently neutralized than the parental virus by the sera collected from the pigs inoculated with C-strain followed by challenge with the Shimen strain (*P < *0.01) (Fig. 7C and D). Our data indicated that the glycosylation at position 986 of the E2 glycoprotein affects its ability to induce NAbs.

## DISCUSSION

**FIG 8:**
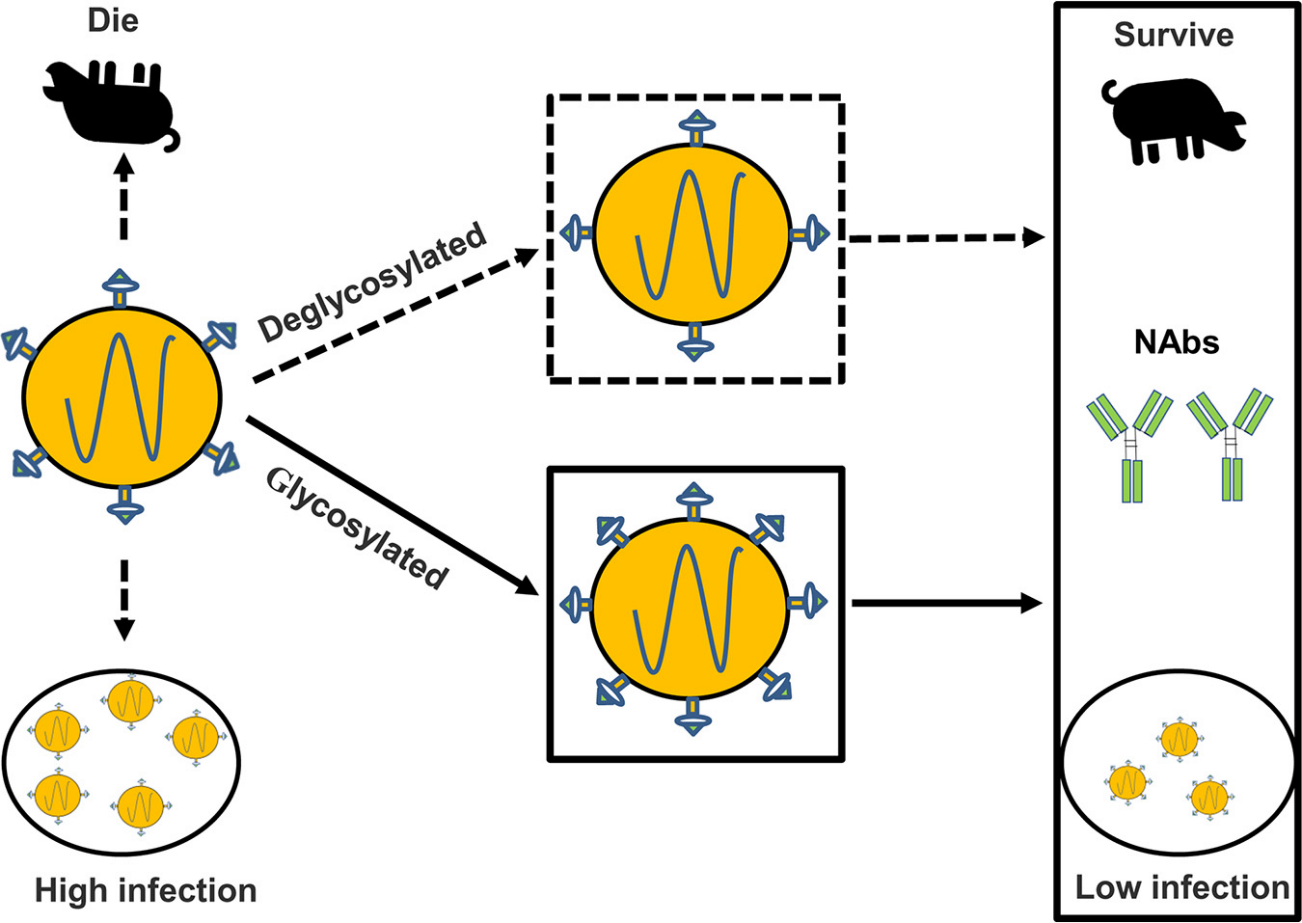
A schematic model of the effects of the unique glycosylation at position 986 on CSFV replication, antigenicity, and pathogenicity. The addition of the unique glycosylation at position 986 to E2 of the highly virulent Shimen strain can influence virus replication, increase neutralizing antibodies, and attenuate the virus in pigs. The dotted line indicates the previous findings on deglycosylation, and the full line indicates the findings on additional glycosylation in the present study.

It has been demonstrated that removing glycosylation at specific sites on the CSFV highly virulent Brescia strain E^rns^, E1, or E2 glycoprotein attenuates virulence and usually protects the pigs from viral challenge. This study first found that the Shimen strain was attenuated by adding a glycan to the E2 glycoprotein. Importantly, the attenuated virus elicited protective immunity. Taken together, our study allows us to present a new model of the effects of glycosylation on viral infectivity, virulence, and antigenicity (Fig. 8).

C-strain is characterized by the induction of fever response and robust replication in the spleen of rabbits. Previously, it has been demonstrated that the 3′-UTR of C-strain played an important role in the fever response in rabbits and the attenuation in pigs (6, 28). This study found that T988A mutation disabled the C-strain to induce fever response in rabbits; meanwhile, the site is responsible for viral attenuation. The molecular determinants for the fever response of C-strain in rabbits could be associated with the virus attenuation in pigs.

Recently, we demonstrated that R132, S133, and D191 in the Domain II of the E2 are involved in CSFV virulence (27). In addition, we showed that P108 and T109 on the E2 glycoprotein Domain I associated with the adaptation of CSFV C-strain to the rabbit. However, the two amino acids are not critical for the virulence of the virus, indicating that the molecular basis for the adaptation and virulence of the CSFV C-strain is different (27). The insertion of 12-nucleotide T in the 3′-UTR is a molecular determinant for the attenuation of C-strain in pigs (28). This study found that the unique glycosylation at position 986 on the E2 glycoprotein Domain IV was associated with the attenuation of CSFV in pigs. Taken together, a novel molecular determinant for C-strain attenuation was identified in this study.

Glycosylation can impact viral interactions with cell surface attachment factors and may impact virion stability and virus replication (8). The ZIKV envelope residues E152/156/158 surrounding a unique *N*-glycosylation site affect the early stages of virus infection in human cells (29). Loss of the glycosylation on the NA of H1N1 influences virus attenuated viral budding and replication (30). It has been shown that the residue at position 830 in CSFV E2 was involved in host tropism and played a role in cell-to-cell spreading (31). In the CSFV Brescia strain, E2 residues 829–837 TAVSPTTLR of CSFV polyprotein play a significant role in CSFV virulence (32), and this region is the minimal domain required for binding to pig antibodies generated during CSFV infection (33). In this study, we proved that the unique glycosylation located in the C-terminal region of E2 is involved in viral spread and virulence.

The glycosylation at position 986 on the E2 changes the electrophoretic mobility of the E1-E2 heterodimers, while Cys on the E1 affects the electrophoretic mobility of the E1-E2 heterodimers (34). Deglycosylation on the E2 of highly virulent CSFV strain Brescia does not affect E2 dimerization in infected cells but alters virulence in pigs (10). However, mutation of specific cysteine residues on the E1 of Brescia strain affects formation of E1-E2 heterodimers in cells and virulence in pigs (34). The *N*-linked glycosylation at the E2-dimer interface is a significant determinant of ZIKV pathogenicity (35), although the effects of the glycosylation on the E2 dimerization are unknown. Interestingly, we showed that the replacement of ^986^NYA^988^ with ^986^NYT^988^ on the E2 of the Shimen strain decreases the E2 dimerization and attenuates the virulence of CSFV.

Viral glycoproteins have been shown to influence infectivity *in vivo* (16), virulence (14), and host immune response (13, 19). A deglycosylation mutant based on the simian immunodeficiency virus SIVmac239 shows robust replication, tightly contained chronic infection, and elicitation of potent immunity against the wild-type strain *in vivo* (19). It has been shown that glycosylation patterns of the highly pathogenic H5N1 NA are critical for increased virulence in chickens (14). Glycosylation at positions 158 to 160 of the HA protein of H5N1 viruses enhanced viral productivity, exacerbated the host response, and contributed to the high pathogenicity of the H5N1 virus in mice (36). ZIKV is an emerging mosquito-borne flavivirus that belongs to the family *Flaviviridae*. The *N*-linked glycosylation of the envelope (E) protein mediates ZIKV virulence (15, 35, 37, 38). The 152/156/158 residues surrounding the unique *N*-glycosylation site on the E protein were not correlated with changes in viral binding onto host cells or cellular responses to viral infection (29). The mutants MR766 ZIKV bearing a mutation (N154A) in the E protein or/and two mutations (N130A and N207A) in NS1 that abolished the glycosylation were fully attenuated, inducing high levels of humoral and cell-mediated immune responses and protecting mice from the lethal challenge (35, 39). In the crystal structure of the bovine viral diarrhea virus (BVDV) E2 glycoprotein, the glycosylation at this position is also noteworthy (40). However, the role of glycan in BVDV pathogenicity remains unclear. In the present study, we have shown that the glycosylation at position 986 plays a major role in the induction of NAbs and the attenuation of C-strain. The addition of the glycosylation to E2 of the Shimen strain induces to a higher neutralizing titer. In contrast to the Shimen infection, infection with the attenuated rShimen-A988T may not lead to a significant B-cell depletion, which could also explain the observation. It will be investigated that the additional *N*-glycosylation site renders E2 into a more potent antigen in the future.

C-strain is able to provide complete protection of pigs of almost all ages against lethal challenge (7), and it can confer complete protection as early as 5 dpi (41, 42). In our study, as C-strain is safe for pigs, the mutant rHCLV-T988A revealed no pathogenicity in pigs. However, rHCLV-T988A did not provide complete protection from challenge at 14 dpi in pigs, indicating that the glycosylation at site 988 is essential for the protection elicited by C-strain. Furthermore, it has been shown that the introduction of the group 2 glycan at Asn38_HA1_ to a group 1 stem-nanoparticle (gN38 variant) based on A/New Caledonia/20/99 (H1N1) broadens antibody responses to cross-react with group 2, indicating that glycan repositioning may be a step toward achieving cross-group influenza protection (20). C-strain can confer protection against all the genotypes of CSFV, which could be due to the unique glycosylation at position 986. Remarkably, we first demonstrated that the site played an important role in the protection by C-strain. Both rShimenA988T and rHCLV-T988A are able to induce protection against the challenge, since viremia is undetectable for both groups at all the time points tested. However, while animals infected with rShimenA988T remained clinically protected from challenge, those infected with rHCLV-T988A developed some clinical signs and lesions in internal organs. This difference may be due to the significant replication levels of rShimenA988T and rHCLV-T988A in the spleens.

In summary, we investigated the roles of the glycosylation at position 986 on the E2 glycoprotein in the attenuation and protection of CSFV in pigs. Our findings highlight an important contribution of the unique glycosylation on the E2 to the attenuation and protection of CSFV in pigs. To our best knowledge, this study represents the first report to show the direct association between the addition of glycosylation at a specific site and the reduced virulence of CSFV. Moreover, the significant protective immunity elicited by rShimen-A988T indicated that glycosylation of E2 could be added for the targeted and rapid development of live attenuated vaccines, which provides insights into rational design for CSF vaccines.

## MATERIALS AND METHODS

### Ethics statement.

Animal experiments were conducted using research protocols approved by the Committee on the Ethics of Animal Experiments of Harbin Veterinary Research Institute (HVRI) of the Chinese Academy of Agricultural Sciences (CAAS) (approval numbers: SY-2020-RA-001 for rabbit experiment and SY-2021-SW-020 for virulence study, SY-2019-SW-034 for protection study in pig experiments). The animal experiments were approved by the Committee on the Ethics of HVRI of CAAS with the license SYXK (Heilongjiang) 2011022. It should be noted that animals developing CSF signs were euthanized as soon as they fit the criteria described in the corresponding IACUC protocols.

### Cells and viruses.

SK6 or PK-15 cells were cultured with Dulbecco’s modified Eagle’s medium (DMEM) (cat. no. C11995500BT; Gibco) supplement with 5% heat-inactivated fetal bovine sera (cat. no. 10099-141C; Gibco) at 37°C incubators with 5% CO_2_. Primary swine macrophages were prepared as described previously (43). The CSFV C-strain (GenBank accession no. AY805221), the Shimen strain (GenBank no. AF092448.2), and the mutants were propagated in SK6 or PK-15 cells.

### Generation of CSFV mutants.

The infectious cDNA clone pShimen-A988T was constructed using fusion PCR with the primers described in Table 2. Two fragments containing the mutation amplified from pShimen using the primers pShimen-A988T-1S/1R and pShimen-A988T-2S/2R, respectively. The two PCR products were fused with primers pShimen-A988T-1S/2R. Both fusion PCR products and pShimen were digested using XhoI and BamHI restriction enzymes, and then linked with T4 DNA ligase (cat. no. M0202S; New England BioLabs). A PCR product harboring an NsiI restriction site was amplified using the primers pHCLV-T988A-S/R listed in Table 2. The pHCLV-T988A was constructed by recombining purified PCR product and the infectious clone pHCLV of C-strain digested with NsiI. pShimen-A988T and pHCLV-T988A were validated by PCR, enzyme digestion, and sequencing.

**TABLE 2:** Primers used in this study

Primers	Sequences (5′–3′)
pHCLV-T988A-S	TGCATTTTGGCAAATGAGAC
pHCLV-T988A-R	CTTCAAAGTTTTTGCGTAGTTGAATGTAC
pShimen-A988T-1S	CCACCTCGAGATGCTATGTG
pShimen-A988T-1R	CAAAGTTTTTGTGTAGTTGAATGTACAG
pShimen-A988T-2S	CTGTACATTCAACTACACAAAAACTTTG
pShimen-A988T-2R	GCTTTGTTAACTCGTAAGTGGGCAG
E2^HCLV^T988A-1S	CGGAATTCGCCACCATGGTATTAAGGGGACAGATCGTGC
E2^HCLV^T988A-1R	GTTCTTCAAAGTTTTTGCGTAGTTGAATGTACAG
E2^HCLV^T988A-2S	CTGTACATTCAACTACGCAAAAACTTTGAAGAAC
E2^HCLV^T988A-2R	CGCTCGAGTCAGGCGGAGCCGCCCGCATCCAGGTCAAACCAGTAC
E2^Shimen^A988T-1S	CGGAATTCGCCACCATGGTATTAAGAGGGCAGATCG
E2^Shimen^A988T-1R	GTTCTTCAAAGTTTTTGTGTAGTTGAATGTACAG
E2^Shimen^A988T-2S	CTGTACATTCAACTACACAAAAACTTTGAAGAAC
E2^Shimen^A988T-2R	CGCTCGAGTCAGGCGGAGCCGCCCACGTCCAGGTCAAACCAGT

Note: The mutations are indicated by the underline.

Two mutants were generated as described previously (6). SK6 cells cultured in a 6-well plate were transfected by a mixture containing six micrograms of each infectious clone and 6 μl of X-tremeGENE HP DNA transfection reagent (cat. no. 6366546001; Roche). The transfected cells were passaged several times and the harvested cells were used to detection of the CSFV E^rns^ glycoprotein by a CSFV antigen test kit (cat. no. 99-40939; IDEXX). The collected samples were determined using RT-PCR, sequencing, and IFA.

### Western blotting.

HEK-293T cells seeded in 6-well plates were transfected with the plasmids pE2^Shimen^, pE2^HCLV^, pE2^Shimen^A988T, and pE2^HCLV^T988A, respectively. At 48 hours posttransfection, the transfected cells were lysed by RIPA buffer, and following a gentle agitation on a Fisher Scientific Mini-Tube Rotator for 1 h at 4°C. The cell debris was then removed by centrifuging for 10 min at 10,000 *g* at 4°C. Lysate aliquots were treated with endoglycosidase H according to the manufacturer’s protocols (cat. no. P0702S; New England BioLabs).

For Western blotting, target proteins were separated in 10% sodium dodecyl sulfate (SDS-PAGE) and transferred onto a polyvinylidene difluoride (PVDF) membrane using a semidry transfer system (Bio-Rad, Hercules, CA, USA). The membrane was then blocked in PBST buffer (0.05% Tween 20) supplemented with 5% skim milk for 2 h at room temperature, subsequently, incubating with anti-E2 antibodies (1:1,000 dilution) for 2 h at 37°C. After three washes with PBST buffer, the blots were probed with goat anti-mouse HRP-conjugated secondary antibody (1:5,000 dilution) in PBST buffer with 5% skim milk for 2 h at 37°C. Finally, the bands were visualized in an Odyssey Infrared Imaging System (LI-COR Biosciences) after washing with PBST buffer.

Formations of E2 homodimers and E1-E2 heterodimers by the mutant virus rShimen-A988T and parental virus Shimen strain were analyzed in lysates of SK6-infected cells by Western blotting. The E2 glycoprotein was detected with anti-E2 MAb WH303. SK6 monolayers were infected (MOI = 1) with rShimen-A988T or the parental virus Shimen strain, or were mock infected, harvested at 48 hpi using the lysis buffer (Invitrogen), and incubated at 70°C for 10 min. The samples were run under the nonreducing conditions in 10% SDS-PAGE.

### RT-qPCR.

According to the manufacturer’s protocols, the total RNA of tissues or cells was extracted using RNAiso Plus (cat. no. 9109; TaKaRa). The cDNA was synthesized in a 20-μl volume with avian myeloblastosis virus reverse transcriptase XL (cat. no. 2621; TaKaRa). The copy numbers of CSFV genome were quantified using RT-qPCR (25).

### IFA and virus titration.

SK6 cells seed in 96-well plates were infected with serially diluted mutant viruses or parental viruses and cultured in an 37°C incubator for 48 h. Cold absolute ethanol was used for fixing the cells at −20°C for 20 min. The fixed cells were washed three times with PBS and incubated with an anti-E2 polyclonal antibody (PAb) at 37°C for 2 h. After washing with PBS five times, the cells were incubated with Alexa Fluor 488 goat anti-rabbit IgG (cat. no. A11034; Roche) 37°C for 1 h and then washed five times with PBS. The cells were analyzed for green fluorescence using an inverted fluorescence microscope. According to the Reed-Muench method, the virus titers were calculated and expressed as median tissue culture infective doses per milliliter (ml).

### Virus growth kinetics.

To analyze the growth characteristics of the mutants, SK6 cells were infected in triplicate with mutant viruses or parental viruses at a multiplicity of infection (MOI) of 0.01. Two hours after incubation at 37°C, the cells were washed with 200 μl of phosphate-buffered saline (PBS), and fresh DMEM containing 2% FBS was added. Supernatants were harvested at the indicated time points, and the virus titers were determined by IFA in SK6 cells and calculated using the Reed-Muench method.

### Focus formation assay.

SK6, PK-15 cells, or porcine primary macrophages were seeded in Lab-Tek II chamber slides (Thermo Fisher Scientific, Waltham, MA) and infected with parent and mutant virus rShimen-A988T at an MOI of 0.001, respectively. After incubation at 37°C for 1 h, the cells were washed once with PBS, overlaid with MEM containing 0.295% TPB, 1% Bacto agar (Becton, Dickinson), and 10% fetal bovine sera, and incubated for 48 h at 37°C in the presence of 5% CO_2_. After the incubation, the cells were fixed with 100% cold acetone for 10 min and incubated for 2 h with the MAb WH303 against E2. The cells were then washed with PBS and incubated for 1 h with a fluorescein isothiocyanate (FITC)-conjugated goat anti-mouse IgG (cat. no. AP124F; Merck-Millipore). After incubation with the conjugate, the cells were washed again, and the E2-positive foci of the infected cells were determined using a fluorescence microscope (Axiovert 200; Carl Zeiss, Oberkochen, Germany). The number of the infected cells per focus was counted for 30 independent foci (*n *= 30), and the statistical significance was calculated using the *t* test.

### Serum-virus neutralization test.

Neutralization assay was performed as described previously (26). Briefly, (i) diluted serum samples of pigs were incubated with 100 TCID_50_ of the CSFV Shimen strain or mutant; and (ii) 10-fold-diluted serum samples were mixed with 10^2^, 10^3^, or 10^4^ TCID_50_ of the CSFV Shimen strain or mutant rShimen-A988T. After inoculation at 37°C for 1 h, 100 μl of the mixture was added to PK-15 cells cultured in 96-well plates. Two hours after incubation at 37°C, the cells were washed once with DMEM and cultured in DMEM containing 2% FBS for 48 h. The neutralizing activity of the sera was tested by IFA in the virus-infected cells. Serum neutralizing titers are expressed as 50% neutralizing dilution (ND_50_).

### Experimental infection of rabbit with the mutant rHCLV-T988A.

Fourteen 12-week-old New Zealand white rabbits were used in this study. The rabbits were housed in Harbin Veterinary Research Institute and randomly allocated to three groups. After an acclimatization period, the rabbits were inoculated intravenously via the marginal ear vein with 10^4^ TCID_50_ of viruses shown in Table 1. Six rabbits in group 1 each were vaccinated with the mutant virus rHCLV-T988A, four rabbits in group 2 each were injected with C-strain vaccine (positive control) or DMEM (group 3, negative control). To monitor the fever response, the rectal temperatures of all the rabbits were recorded every 6 h from 24 to 72 hpi. At 3 dpi, half of the rabbits selected randomly from each group were euthanized, and the total RNA of CSFV in these spleens was extracted and determined by RT-qPCR as described previously (25). All serum samples from the rabbits were collected at 7 dpi to test the antibodies against the CSFV E2 protein by the IDEXX HerdCheck CSFV Ab ELISA following the manufacturer’s instructions (cat. no. 99-43220; IDEXX).

### Experimental infection of pigs with the mutants.

To evaluate the effect of the glycosylation on virulence, three groups of pigs were inoculated with 10^4^ TCID_50_ of the parental virus Shimen strain and mutants. A total of nine 5-week-old SPF pigs were randomly divided into 3 groups (Groups 1–3, *n *= 3), and each group was housed in an individual room. The pigs in Group 1 were inoculated i.m. with rHCLV-T988A; the animals in Group 2 were inoculated i.m. with rShimen-A988T; and the pigs in group 3 were infected with the Shimen strain serving as a positive control to assess the pathogenicity.

To evaluate the protective efficacy of the mutants, eight pigs were randomly allocated into 3 groups. The pigs in Groups A and B (*n *= 3) were inoculated with 10^4^ TCID_50_ of rHCLV-T988A or rShimen-A988T, while the pigs in Group C (*n *= 2) were mock-infected. At 14 dpi, the inoculated pigs were challenged with 10^5^ TCID_50_ of the Shimen strain.

Clinical signs and rectal temperatures were monitored daily (26), and the serum samples and anticoagulation blood samples of pigs were collected at 3, 7, 10, and 14 dpi or dpc. E2-specific antibodies were measured with the IDEXX HerdCheck CSFV Ab ELISA according to instructions (cat. no. 99-43220; IDEXX). Anticoagulated blood samples were also collected at 3, 7, 10, and 14 dpi or dpc and subjected to RT-qPCR to determine the viral RNA copies as described above.

### Statistical analysis.

Differences between groups were analyzed for statistical significance using the Student's *t* test by GraphPad Prism 7. An unadjusted *P value* of less than 0.05 was considered to be significant.
